# In-hospital cardiac arrest due to acute pulmonary embolism: a case report of successful catheter-directed thrombectomy on a patient with VA-ECMO

**DOI:** 10.1093/ehjcr/ytaf342

**Published:** 2025-08-25

**Authors:** Merve Kural, Stephan Rosenkranz, Stephan Baldus, Alexander Christian Bunck, Tobias Tichelbäcker

**Affiliations:** Department of Cardiology, Pulmonology and Intensive Care Medicine, Heart Center, University of Cologne, Kerpener Str. 62, Cologne 50937, Germany; Department of Cardiology, Pulmonology and Intensive Care Medicine, Heart Center, University of Cologne, Kerpener Str. 62, Cologne 50937, Germany; Department of Cardiology, Pulmonology and Intensive Care Medicine, Heart Center, University of Cologne, Kerpener Str. 62, Cologne 50937, Germany; Department of Radiology, University of Cologne, Kerpener Str. 62, Cologne 50937, Germany; Department of Cardiology, Pulmonology and Intensive Care Medicine, Heart Center, University of Cologne, Kerpener Str. 62, Cologne 50937, Germany

**Keywords:** Case report, Pulmonary embolism, Catheter-directed thrombectomy, VA-ECMO, Cardiac arrest

## Abstract

**Background:**

First-line therapy for high-risk pulmonary embolism (PE) is systemic thrombolysis. Catheter-directed thrombectomy (CDT) poses as a secondary option, primarily in patients with contraindications for systemic thrombolysis. However, in patients with haemodynamic instability or cardiac arrest, CDT can worsen the haemodynamic situation making use of large thrombectomy catheters. The implementation of extracorporeal life support such as veno-arterial extracorporeal membrane oxygenation (VA-ECMO) can play a decisive role in making CDT possible. Herein, we present a case of CDT on a high-risk PE patient under VA-ECMO.

**Case summary:**

A 73-year-old White male was hospitalized in order to perform abdominal surgery. Afterwards, multiple complications led to recurring operations and a prolonged immobilization time. In the aftermath, the patient suffered an in-hospital cardiac arrest and was put on VA-ECMO. A computed tomography pulmonary angiography presented bilateral central PE. Due to contraindications for systemic thrombolysis, successful CDT using a FlowTriever catheter was performed, leading to a reduction of mean pulmonary arterial pressure. ECMO therapy could be terminated in the following days. The patient was eventually discharged without any signs of right heart strain in transthoracic echocardiogram, neurological sequelae or dyspnoea.

**Discussion:**

According to current ESC-guidelines, first-line therapy for high-risk PE is systemic thrombolysis, and CDT is a secondary option. In our case, CDT under VA-ECMO was feasible and led to a rapid improvement in haemodynamics, resulting in a long-term recovery. Thus, the definite significance of CDT has yet to be identified, especially concerning PE with refractory cardiac arrest and contraindications for systemic thrombolysis.

Learning points

- According to current ESC-guidelines, catheter-directed thrombectomy (CDT) only poses as a secondary therapeutic option in patients with high-risk PE if contraindications for systemic thrombolysis are given or if systemic thrombolysis has already failed.
- In patients with haemodynamic instability or cardiac arrest, CDT under the implementation of VA-ECMO can play a decisive role. In our depicted case, CDT under VA-ECMO was feasible and led to a rapid improvement in haemodynamics, resulting in a long-term recovery of our patient with no signs of sequelae.

## Introduction

Pulmonary embolism (PE) is a cardiovascular disease with a high mortality and an increase in its incidence in the last years. It can be classified according to its early mortality risk (in-hospital or 30-day-mortality) into high-, intermediate-high-, intermediate-low-, or low-risk as per current European Society of Cardiology (ESC) guidelines, and treatment strategies depend on the particular risk class. High-risk PE requires haemodynamic instability, which includes cardiac arrest, obstructive shock, or persistent hypotension.^1^

There is a wide range of therapeutic options concerning PE extending from mere anticoagulatory treatment to the additional use of systemic thrombolysis, catheter-directed thrombolysis, catheter-directed thrombectomy (CDT) or surgical treatment.^1^ According to current ESC guidelines, first-line therapy for high-risk PE is systemic thrombolysis, and CDT is considered a secondary option if systemic thrombolysis is contraindicated or has failed.^1^

Regarding the most common risk factors for PE, some of those depict contraindications for systemic thrombolysis themselves, such as recent surgery and central nervous neoplasms.^1^ Hence, there is a relevant number of patients presenting with high-risk PE and contraindications for systemic thrombolysis. In these cases, CDT appears to be a feasible and safe^2^ treatment strategy.

However, CDT requires large thrombectomy catheters to cross the tricuspid^3^ and pulmonary valve.^4^ This may lead to haemodynamic instability and worsening of obstructive shock, especially in high-risk patients. Yet, the use of CDT is valuable in high-risk PE with contraindications for systemic thrombolysis. In such cases, the implementation of extracorporeal life support (ECLS) such as veno-arterial extracorporeal membrane oxygenation (VA-ECMO) can play a decisive role in making CDT possible.

Similarly, patients with cardiac arrest due to PE but no return of spontaneous circulation (ROSC) must be considered. With PE as a potentially reversible cause for cardiac arrest, these patients are often well eligible for VA-ECMO as a bridge to reperfusion treatment. However, the implementation of VA-ECMO on a patient with systemic thrombolysis poses an increased risk of femoral bleeding. Thus, a patient presenting with PE and no ROSC is rather suitable for CDT as well.

For these reasons, the use of CDT is becoming more common in clinical practice, particularly for patients with high-risk PE who have contraindications to systemic thrombolysis. The combination of VA-ECMO and CDT may offer a therapeutic option in selected high-risk patients, especially when haemodynamic instability could be exacerbated by the procedure itself. Herein, we present a case of CDT on a high-risk PE patient under VA-ECMO.

## Summary figure

**Figure:**
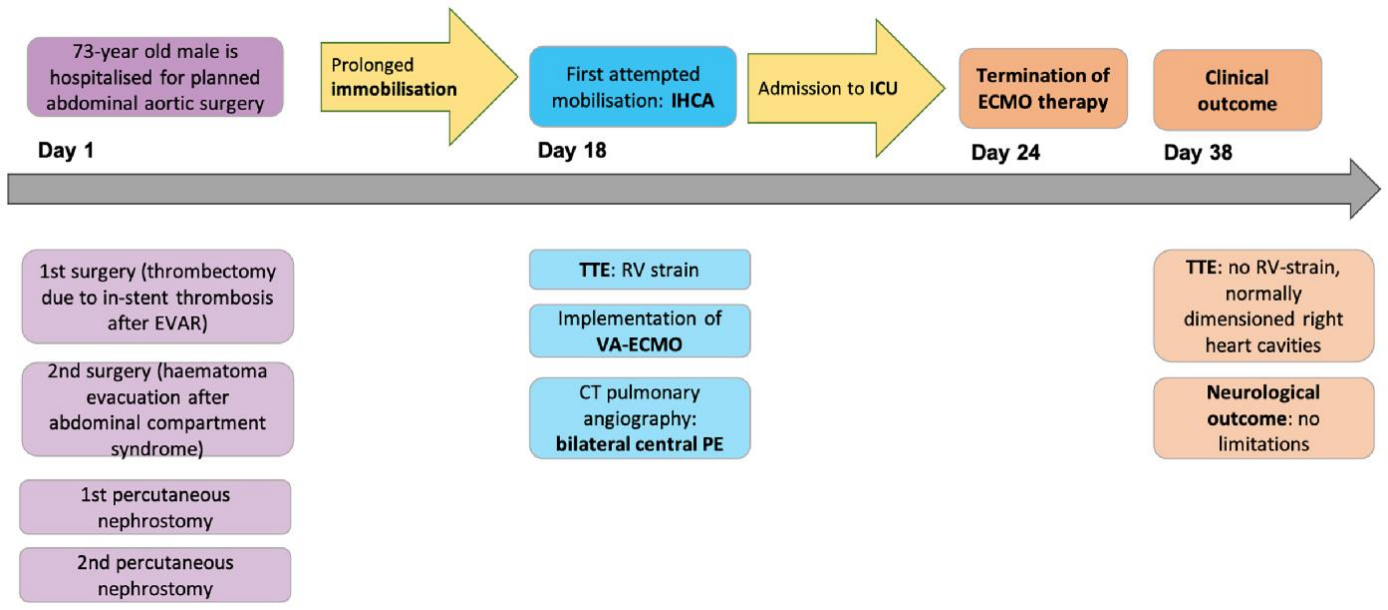
Illustration of the timeline of hospitalization and key interventions. EVAR, endovascular aneurysm repair; IHCA, in-hospital cardiac arrest; TTE, transthoracic echocardiography; VA-ECMO, veno-arterial extracorporeal membrane oxygenation; CT, computed tomography; PE, pulmonary embolism; ICU, intensive care unit; RV, right ventricle.

## Case presentation

The hospitalization of the 73-year-old White male patient occurred in order to perform abdominal surgery due to in-stent thrombosis after endovascular aneurysm repair of the abdominal aorta. On admission, physical examination was unremarkable, and laboratory results showed normal renal function, a normal complete blood count, including platelet count, and normal activated partial thromboplastin time (aPTT) and international normalized ratio (INR). Surgery was performed on hospital day 5. Afterwards, the patient experienced retroperitoneal bleeding and abdominal compartment syndrome which required surgical revision and hematoma evacuation on hospital day 6. As a consequence, an intra-operative ureteral injury occurred, making percutaneous nephrostomy on hospital day 6 necessary. The nephrostomy catheter was malpositioned, so that an interventional correction on hospital day 16 was required. Consequently, recurring operations and interventions led to a prolonged immobilization time, and the patient was prophylactically anticoagulated via continuous intravenous infusion of unfractionated heparin, with the infusion rate adjusted to maintain a target aPTT of 40–50 s.

In terms of other relevant pre-existing conditions, peripheral arterial occlusive disease and gout were known. Furthermore, the patient featured a cardiovascular risk profile with hyperlipidaemia, overweight and high blood pressure.

During the first attempt of mobilization on hospital day 24, the patient suffered an in-hospital cardiac arrest (IHCA) and cardio-pulmonary resuscitation (CPR) was performed. Physical examination revealed no signs of bleeding or trauma. Transthoracic echocardiogram on-site revealed right heart strain and a thrombus of the right atrium and the inferior vena cava. These findings, combined with risk factors such as repeated surgery and extended immobilization, were indicative of an acute PE as a cause for IHCA.

Due to the lack of ROSC and suspected PE representing a potentially reversible cause, the patient was put on VA-ECMO after 38 min owing to poor vascular conditions.

**Figure 1:**
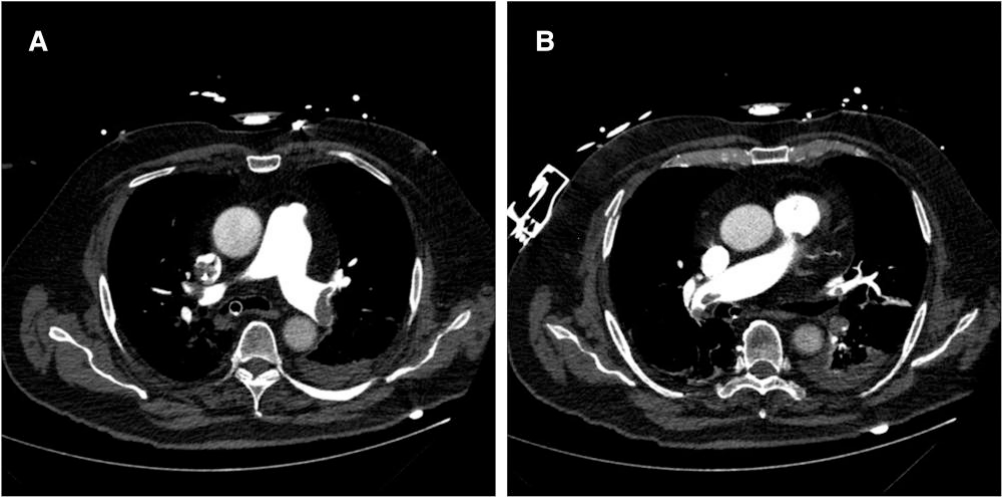
(*A*) Axial slice of computed tomography pulmonary angiography (CTPA) visualizing central thrombotic sub-total occlusion of the right and left pulmonary artery (PA). (*B*) Axial slice of CTPA visualizing central sub-total thrombotic occlusion of the right PA.

**Figure 2:**
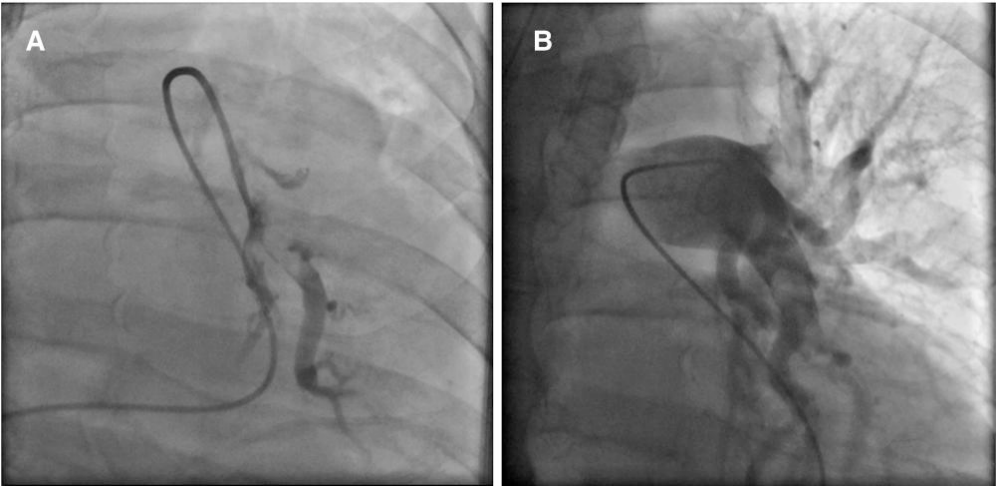
(*A*) Pulmonary angiogram exemplarily of the left pulmonary artery (PA) showing sub-total thrombotic obstruction. (*B*) Re-establishment of blood flow after catheter-directed thrombectomy in the left PA.

A subsequently performed computed tomography pulmonary angiography (CTPA) presented a central, segmental and sub-segmental PE on both sides (*Figure 1*). The pulmonary embolism severity index was 193 points, the troponin T level was elevated (1.16 µg/L), and according to ESC guidelines the classification based on the early mortality risk was high-risk.^1^ Based on multiple contraindications for systemic thrombolysis due to surgery with bleeding complications and the implementation of VA-ECMO, an interventional thrombectomy using a FlowTriever catheter was performed (*Figure 2*). Due to the venous ECMO cannula which was placed in the right femoral vein, the thrombectomy catheter was inserted in the left femoral vein. Doing so, thrombotic material from both pulmonary arteries (PA) could successfully be aspirated making use of Mother-and-Child thrombectomy technique, meaning the advancement of a smaller (16 French) into a larger catheter (24 French).^5^ Mean PA pressure was instantly reduced from 39 to 31 mmHg. After CDT, laboratory results showed an improved but still elevated creatinine of 1.67 mg/dL (related to recent ureteral injury and nephrostomy), with normal platelet count and INR, and elevated aPTT under ongoing heparin therapy.

Subsequently, the patient was admitted to intensive care unit. Catecholamines could rapidly be tapered and weaning from mechanical ventilation was successful in the following as well. ECMO therapy was terminated 6 days after initiation, following the achievement of ROSC and recovery of sufficient cardiac function.

**Figure 3:**
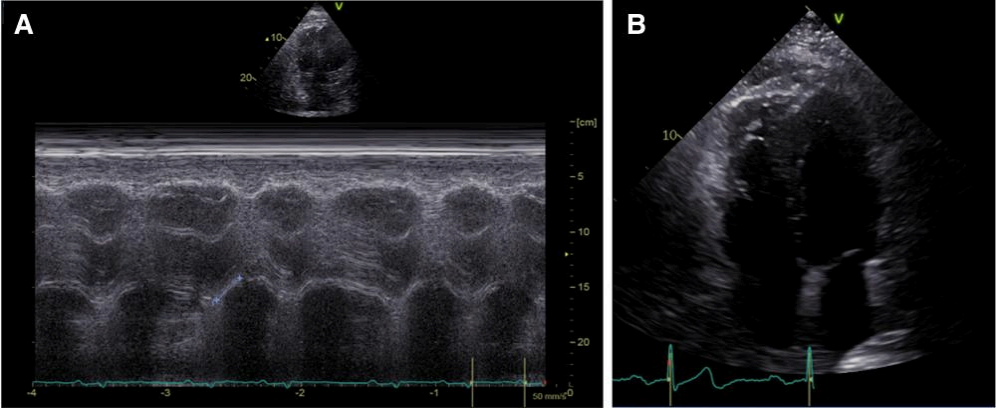
(*A*) Normal tricuspid annular plane systolic excursion (TAPSE) of 20 millimetres before discharge. (*B*) 4-Chamber-View with no signs of dilatation of the right heart cavities in a 1-year-follow-up.

In the aftermath, transthoracic echocardiogram 20 days after the event showed no abnormalities, a normal right ventricular (RV) systolic function such as normally dimensioned right heart chambers (*Figure 3*). The patient was eventually discharged without any neurological limitations or dyspnoea. In a 1-year-follow-up, the patient reported no signs of dyspnoea or neurological sequelae. The N-terminal pro–B-type natriuretic peptide (NT-proBNP) level was within the normal range for age.

## Discussion

According to current ESC guidelines, first-line therapy for high-risk PE is systemic thrombolysis, and CDT is only a secondary therapeutic option.^1^

In a study on intermediate high-risk PE-patients receiving systemic thrombolysis, bleeding complications occurred in 12%, whilst bleeding complications in patients only receiving anticoagulatory treatment occurred in 2.5% in the 30-day follow-up.^6^ Systemic thrombolysis is associated with a significantly increased risk of bleeding complications, which, in patients already receiving VA-ECMO or with recent surgical bleeding, limits its safety and feasibility. Thus, in our case, an alternative therapeutic option was crucial.

Existing evidence suggests that CDT is a safe therapeutic option that can rapidly improve haemodynamics and RV function, particularly in intermediate-high risk PE patients.^7^ In the prospective FLAME study published in 2023, CDT in high-risk PE was associated with a low in-hospital mortality of <2% and clinically relevant adverse outcomes in 17%, the latter one being significantly lower than the performance goal.^8^ A prospective study published in 2023 showed no procedure-related major adverse events and no deaths during 30-day follow-up in intermediate-high and high-risk PE.^2^ Also, a reduction of RV to left ventricular (LV) ratio and an increase of tricuspid annular plane systolic excursion (TAPSE) before discharge could be seen. While these findings are promising, the current evidence base for CDT remains strongest in intermediate-high risk PE. Further studies are needed to confirm the safety and efficacy of CDT, particularly in high-risk patients and in those supported with VA-ECMO.

The feasibility and safety of CDT under VA-ECMO need further evaluation. A preliminary, single-centre study on high-risk PE with CDT due to contraindications for thrombolysis under VA-ECMO support (or standby) was published in 2023.^9^ Herein, 15 patients were included. The primary outcome (recurrent PE, heart failure hospitalization, and all-cause death at 30 days) emerged in five patients (33.3%). There was one periprocedural death. These results suggest a practicality concerning VA-ECMO in high-risk PE with CDT, especially regarding safety concerns.

Still, in patients receiving ECLS, particularly VA-ECMO, PA pressure measurements can be less reliable due to the reduction of right ventricular preload. As the ECLS assists in maintaining systemic circulation, PA pressures may not accurately reflect the true haemodynamic status of the patient. This limitation should be considered when interpreting PA pressures.

In our depicted case, CDT under VA-ECMO was feasible and led to a rapid improvement in haemodynamics, resulting in a long-term recovery of our patient with no signs of sequelae. This case supports the feasibility of CDT under VA-ECMO in selected high-risk PE patients with contraindications to systemic thrombolysis. However, the safety and efficacy of this approach require further evaluation in larger, controlled studies.

Summed up, according to current ESC-guidelines, the first-line therapy for high-risk PE is systemic thrombolysis. Still, the definite significance of CDT has yet to be identified, especially concerning high-risk PE with refractory cardiac arrest and contraindications for systemic thrombolysis.

## Lead author biography


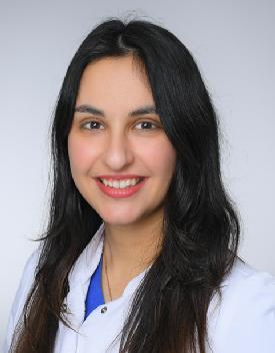


Ms Kural graduated from high school in 2014. She completed medical studies at the Medical Faculty of the University of Cologne in Germany in 2021. Since June 2021, she is employed at the Clinic III for Internal Medicine of the University Hospital Cologne.

## Author contributions

Merve Kural (Conceptualization, Data curation, Visualization, Writing—original draft, Writing—review & editing), Stephan Rosenkranz (Conceptualization, Project administration, Resources), Stephan Baldus (Project administration, Resources), Alexander Christian Bunck (Investigation, Resources), and Tobias Tichelbäcker (Conceptualization, Investigation, Project administration, Supervision, Writing—review & editing)

**Consent:** Written informed consent was obtained from the patient in accordance with COPE guidelines.

**Funding:** No specific funding was received for this case report.

## Data Availability

All relevant data underlying this case report are included in the article. No additional data are available.
